# Development and evaluation of a nomogram prediction model for invasion and metastasis in primary liver cancer based on serum CD147 and IL-6

**DOI:** 10.3389/fonc.2025.1524765

**Published:** 2025-08-21

**Authors:** Wei Zhang, Di Wu, Xinping Wang, Hua Zhang, Ming Yu

**Affiliations:** ^1^ Department of Digestive Surgery, Xi’an People’s Hospital (Xi’an Fourth Hospital), Xi’an, China; ^2^ Department of Hematology, The First Affiliated Hospital of Xi’an Jiaotong University, Xi’an, China

**Keywords:** CD147, IL-6, primary liver cancer, invasion and metastasis, nomographic prediction model

## Abstract

**Objective:**

This study aims to develop a prediction model for invasive metastasis of primary liver cancer based on serum extracellular matrix metalloproteinase-inducing factor (CD147) and interleukin-6 (IL-6).

**Methods:**

Between July 2022 and August 2024, 170 surgically treated primary hepatocellular carcinoma patients at our hospital were recruited. They were divided into a training group (*n* = 120) and a validation group (*n* = 50) at a 7:3 ratio. Univariate and multivariate logistic regression analyses were applied in the training group to identify factors related to invasive metastasis. A risk factor-based bar chart prediction model was then constructed and internally tested. Its goodness of fit was evaluated, and the model’s diagnostic efficacy was assessed using the ROC curve. Finally, decision curve analysis (DCA) was performed to evaluate the model’s clinical value.

**Results:**

In the training group, compared with the noninvasive metastasis group, patients in the invasive metastasis group had a significantly lower percentage of intact envelope and tumor size ≥5 cm, and significantly higher serum alpha-fetoprotein (AFP), alkaline phosphatase (ALP), C-reactive protein/albumin ratio (CAR), oncoglobulin (CEA), CD147, and IL-6 levels (all *p* < 0.05). After logistic multifactorial analysis, intact envelope, tumor > 5 cm, AFP, CAR, CD147, and IL-6 were identified as independent influencing factors for invasive metastasis of primary hepatocellular carcinoma (all *p* < 0.05). A column chart model was constructed. The C-index of the training and validation groups was 0.884 (95% confidence interval [CI]: 0.738–0.932) and 0.841 (95% CI: 0.741–0.939), respectively. The calibration curves showed good agreement between the predicted probability and the actual probability in both the training and validation groups, without significant deviation. The area under the curve (AUC) of the ROC analysis was 0.852 (95% CI: 0.824–0.979) and 0.839 (95% CI: 0.791–0.912), respectively. DCA indicated that the model had clinical application value within a certain range of threshold probabilities.

**Conclusion:**

The prediction model based on serum CD147, IL-6, and other risk factors for the invasion and metastasis of primary hepatocellular carcinoma demonstrates high diagnostic value.

## 1 Introduction

It refers to malignant tumors that originate from the liver, most commonly hepatocellular carcinoma (HCC), in addition to other types of primary liver cancer, such as intrahepatic cholangiocarcinoma (ICC). Primary liver cancer is one of the malignancies with a high incidence worldwide, with high morbidity and mortality rates, particularly in China. According to statistics, its incidence rate ranks sixth, and its mortality rate ranks fourth. In recent years, both its incidence and mortality rates have shown an increasing trend (1). Invasion and metastasis are important factors contributing to the poor prognosis of cancer patients (2). Prolonging the survival time and improving the quality of life of patients with primary liver cancer have gradually become a research focus for clinical workers. Therefore, identifying methods that can predict the risk of invasion and metastasis of patients after treatment and providing timely, targeted intervention are of great significance for improving patient prognosis. Although a variety of factors affecting the invasion and metastasis of primary liver cancer have been evaluated in previous studies—including tumor size, lymph node metastasis, and related biomarkers—an effective and accurate prediction model is still lacking (3, 4). Studies have found that the absence of a tumor capsule is related to regional lymph node metastasis and may be a factor affecting the prognosis of patients with liver cancer (5, 6). In addition, serum tumor markers are important methods for the early diagnosis and monitoring of cancer. Alpha-fetoprotein (AFP), a protein produced by hepatocytes, is also the most widely detected serum biomarker of liver cancer, and excessive AFP is considered to be related to the biological characteristics and burden of more aggressive tumors (7). As a transmembrane glycoprotein, extracellular matrix metalloproteinase-inducing factor (CD147) plays an important role in tumor invasion and metastasis and can degrade the extracellular matrix by promoting the expression and activation of matrix metalloproteinases, thus promoting the invasion and metastasis of tumor cells. Inflammatory response plays an important role in the occurrence and development of tumors. The C-reactive protein/albumin ratio (CAR), which is calculated by dividing C-reactive protein (CRP) by serum albumin (ALB), reflects both the inflammatory and nutritional state of the body. Interleukin-6 (IL-6) is a multifunctional cytokine, and an increased level of IL-6 usually indicates the presence of an inflammatory reaction. It has been reported that serum levels of IL-6, CRP, and other markers are significantly increased in patients with metastatic colorectal cancer, and their combined detection is conducive to predicting cancer mortality after surgery (8, 9). Therefore, monitoring various laboratory indicators in patients with primary liver cancer may play an important role in the early diagnosis of invasion and metastasis.

The nomogram prediction model is a predictive tool that calculates the continuous probability of patient-specific results, which can visually present the results and thus more intuitively predict the risk of those results (10). In this study, the risk factors for invasion and metastasis of primary liver cancer were retrospectively analyzed, and based on this, a nomogram prediction model was constructed to improve the accuracy of diagnosing invasion and metastasis in primary liver cancer, thus providing an important reference for clinical decision-making.

## 2 Data and methods

### 2.1 Study population

A total of 170 patients with liver cancer who underwent surgical treatment in our hospital from July 2022 to August 2024 were selected as the research subjects. After admission, they received targeted treatment until their condition improved and they were discharged. For early-stage patients, the primary treatment options include surgical resection (with a postoperative recovery period of approximately 4–6 weeks), liver transplantation (which typically involves a waiting period of several months), or local ablation (usually administered one to two times per course). Patients with advanced disease were treated with transarterial chemoembolization (TACE) (repeated every 6–8 weeks); targeted combined immunotherapy (atezolizumab + bevacizumab, continued until disease progression or intolerance, with a median duration of 8–12 months); single-agent targeted therapy (lenvatinib/sorafenib, continued for a median of 5–7 months); or second-line therapy (regorafenib, median duration 3–5 months). The patients were divided into a training group (*n* = 120) and a validation group (*n* = 50) in a 7:3 ratio. The training group was divided into invasive and metastatic groups (37 cases) and noninvasive and metastatic groups (83 cases) based on the pathological results of invasion and metastasis. Inclusion criteria were as follows: (1) all patients met the diagnostic criteria for primary liver cancer (11) and were confirmed by pathological and imaging examinations; (2) Child–Pugh liver function classification of grade A or B before surgery; (3) complete pathological and relevant laboratory data were available. The exclusion criteria were as follows: (1) abnormal coagulation function or presence of organ failure; (2) complications involving renal or cardiac dysfunction; (3) presence of infectious diseases; (4) coexistence with other malignant tumors. This study was approved by the hospital’s ethics committee, and all patients signed informed consent forms.

### 2.2 Collection of clinical data

Clinical data collected included gender (woman, man), smoking history, drinking history, TNM stage (I + II, III + IV), presence of liver cirrhosis, number of hepatectomies (1~2, ≥3), as well as age, BMI, capsule integrity, and tumor size (≤ 5 cm, > 5 cm).

### 2.3 Laboratory indicator test

An automatic biochemical analyzer (Beckman Coulter, AU5800) was used to detect laboratory-related indicators, including AFP, alkaline phosphatase (ALP), CAR, carcinoembryonic protein (CEA), CD147, and IL-6. AFP (Art. No. JKSJ–2034), ALP (Art. No. JK-(a)-2565), CRP (Art. No. JK-(a)-2334), ALB (Art. No. JK-(a)-2381), CEA (Art. No. JK-SJ-36565), CD147 (Art. No. JK-SJ-32631), and IL-6 (Art. No. JK-(a)-2154) kits were purchased from Shanghai Jingkang Biological Engineering Co. Ltd., and all detection steps were performed strictly in accordance with the manufacturer’s instructions.

### 2.4 Development and evaluation of the nomogram prediction model

In the training set, univariate analysis was first performed on various clinicopathological indicators and protein expression indicators to screen for factors potentially associated with the invasion and metastasis of liver cancer (*p* < 0.05). Subsequently, these factors were included in a multivariate logistic regression analysis to identify independent risk factors. The variance inflation factor (VIF) was applied for multicollinearity diagnosis to ensure that there was no severe multicollinearity among the factors (a VIF < 10 was considered indicative of the absence of severe multicollinearity). A nomogram model was constructed based on the results of the multivariate logistic regression analysis. Scores were assigned to each independent risk factor in the model, and the probability of invasion and metastasis of liver cancer was predicted by calculating the total score. The higher the total score, the greater the predicted likelihood of invasion and metastasis of liver cancer. The diagnostic performance of the model was evaluated using the receiver operating characteristic (ROC) curve, and the area under the curve (AUC), sensitivity, specificity, and other indices were calculated to validate the accuracy and clinical utility of the model. Internal validation was performed using the bootstrap resampling method with 1,000 iterations to assess model stability. For each iteration, 70% of the modeling dataset was randomly sampled with replacement to reconstruct the model, and the remaining 30% was used for validation. The bias-corrected consistency index (C-index) was calculated to adjust for optimism. This process accounted for potential overfitting in the training cohort and provided a more robust estimate of model performance. Meanwhile, decision curve analysis (DCA) was applied to evaluate the clinical application value of the nomogram model and to judge the clinical benefits of the model at different threshold probabilities.

### 2.5 Statistical analysis

Data analysis was conducted using SPSS 26.0 software and R 4.3.1 software. When the measurement data conformed to a normal distribution, they were represented by (S), and comparisons between the two groups were made using the independent sample *t*-test. For data that did not conform to a normal distribution, the median (M; Q1, Q3) was, and the Mann–Whitney *U* test was applied for group comparisons. Categorical data were compared using the *χ* ^2^ test or Fisher’s exact test and were expressed as the number of cases. Multivariate logistic regression analysis was employed to screen the risk factors for gastric cancer invasion and metastasis, and a *p*-value < 0.05 was considered statistically significant. The research subjects were randomly divided into the training and validation sets at a 7:3 ratio using the “caret” package in R software. To assess potential multicollinearity among independent variables, a VIF test was performed. The “rms” package in R software was used to establish the nomogram model, and the “pROC” package was used to draw the ROC curve. The bootstrap method (with 1,000 repeated samplings) was adopted to conduct internal validation of the model and to draw the calibration curve, and the “DCA.r” program was used to draw the decision curve. Significance testing was performed using a two-sided alpha level of 0.05.

## 3 Results

### 3.1 Comparison of clinical data between the training and validation groups

There were no statistically significant differences in clinical data between the training and validation groups (*p* > 0.05). This result indicates that the grouping followed the principle of randomization, which helps to effectively avoid bias caused by uneven distribution of variables between the two groups (**Table 1**).

**Table 1:** Comparison of clinical data between the training and validation groups (cases; %), ( $\bar{x}\pm s$ ).

Group	Training group (*n* = 120)	Validation group (*n* = 50)	χ^2^ */t*	*p*-value
Gender				
Woman	36	20	1.598	0.206
Man	84	30		
Age (years)	58.15 ± 8.61	59.26 ± 7.23	0.801	0.424
BMI (kg/m^2^)	22.04 ± 2.24	21.99 ± 2.05	0.136	0.892
Smoking history	36	16	0.067	0.797
Drinking history	26	10	0.059	0.809
TNM staging				
Stage I + II	102	39	1.222	0.269
Stage III + IV	18	11		
Cirrhosis				
Exist	42	22	1.218	0.270
Nonexistent	78	28		
Number of hepatectomies				
1~2	79	32	0.052	0.819
≥ 3	41	18		
Quantity				
1	100	36	2.833	0.092
≥ 2	20	14		
Capsule intact	20	9	0.044	0.833
Tumor size (cm)				
≥ 5	28	17	2.063	0.151
< 5	92	33		
Child–Pugh				
A	100	40	0.256	0.613
B	20	10		
AFP (ng/mL)	435.52 ± 84.46	438.26 ± 90.42	0.189	0.851
ALP (U/L)	185.25 ± 63.44	178.26 ± 74.06	0.623	0.535
CAR	0.74 ± 0.21	0.77 ± 0.30	0.743	0.458
CEA (μg/L)	11.91 ± 2.39	12.05 ± 3.09	0.318	0.751
CD147 (pg/mL)	171.70 ± 37.78	175.26 ± 40.19	0.549	0.584
IL-6 (μg/L)	2.39 ± 0.45	2.41 ± 0.88	0.196	0.845

### 3.2 Clinical data analysis of two groups of patients in the training group

There were no significant differences in gender, smoking history, drinking history, TNM stage, cirrhosis, number of hepatic resections as a percentage, age, or BMI between patients in the invasive metastasis group and those in the noninvasive metastasis group (*p* > 0.05). However, compared with the noninvasive metastasis group, the proportion of patients with an intact capsule and tumor size < 5 cm was significantly lower in the invasive metastasis group (*p* < 0.05; **Table 2**).

**Table 2:** Clinical data analysis of two groups of patients in the training group (cases; %), ( $\bar{x}\pm s$ ).

Group	Invasion and metastasis group (*n* = 37)	Noninvasive metastasis group (*n* = 83)	*χ* ^2^ */t*	*p*-value
Gender				
Woman	12 (32.43)	24 (28.92)	0.151	0.698
Man	25 (67.57)	59 (71.08)		
Age (years)	57.95 ± 7.82	58.24 ± 8.96	0.170	0.865
BMI (kg/m^2^)	21.82 ± 2.14	22.14 ± 2.28	0.723	0.471
Smoking history	10 (27.03)	26 (31.33)	0.225	0.635
Drinking history	9 (24.32)	17 (20.48)	0.223	0.637
TNM staging				
Stage I + II	31 (83.78)	71 (85.54)	0.062	0.803
Stage III + IV	6 (16.22)	12 (14.46)		
Cirrhosis				
Exist	10 (27.03)	32 (38.55)	1.495	0.221
Nonexistent	27 (72.97)	51 (61.45)		
Number of hepatectomies				
1~2	23 (62.16)	56 (67.47)	0.321	0.571
≥ 3	14 (37.84)	27 (32.53)		
Quantity				
1	30 (81.08)	70 (84.34)	0.195	0.658
≥ 2	7 (18.92)	13 (15.66)		
Capsule intact	1 (2.70)	19 (22.89)	7.510	0.006
Tumor size (cm)				
≥ 5	22 (59.49)	6 (7.23)	39.027	< 0.001
< 5	15 (40.54)	77 (92.77)		
Child–Pugh				
>A	30 (81.08)	70 (84.34)	0.229	0.632
B	7 (18.92)	13 (15.66)		

### 3.3 Analysis of serum laboratory indicators in two groups of patients in the training group

Compared with the noninvasive metastasis group, serum AFP, ALP, CAR, CEA, CD147, and IL-6 levels were significantly increased in the invasive metastasis group (*p* < 0.05) in **Table 3**.

**Table 3:** Analysis of serum laboratory indicators in the two patient groups in the training group ( $\bar{x}\pm s$ ).

Group	Invasion and metastasis group (*n* = 37)	Noninvasive metastasis group (*n* = 83)	*χ* ^2^ */t*	*p*-value
AFP (ng/mL)	527.23 ± 111.64	394.63 ± 72.35	7.777	< 0.001
ALP (U/L)	207.30 ± 68.29	175.42 ± 61.28	2.540	0.012
CAR	1.02 ± 0.15	0.62 ± 0.24	9.345	< 0.001
CEA (μg/L)	15.02 ± 4.43	10.52 ± 1.49	8.296	< 0.001
CD147 (pg/mL)	214.96 ± 45.15	152.41 ± 34.49	9.643	< 0.001
IL-6 (μg/L)	2.96 ± 0.53	2.14 ± 0.41	9.218	< 0.001

### 3.4 Multivariate analysis of invasion and metastasis of primary liver cancer

The nondependent variables of whether the patient had invasion and metastasis (0 = nonoccurrence, 1 = occurrence) were taken as the independent variables. Statistically significant indicators identified in **Tables 1**, **2** were selected as the independent variables. Continuous variables were analyzed using their actual measured values, while categorical variables were assigned values for analysis (capsule intact: 1 = No, 0 = Yes; tumor size: 1 = ≥ 5 cm, 0 = < 5 cm). Logistic multivariate analysis showed that intact capsule, tumor > 5 cm, AFP, CAR, CD147, and IL-6 were all independent factors affecting the invasion and metastasis of primary liver cancer (*p* < 0.05) (**Table 4**). To assess potential multicollinearity among independent variables (AFP, CD147, IL-6), a VIF test was performed. Results showed that all VIF values were below the threshold of 10, with specific values as follows: AFP (VIF = 3.21), CD147 (VIF = 2.89), IL-6 (VIF = 3.57), capsule integrity (VIF = 1.82), tumor size (VIF = 2.11), and CAR (VIF = 2.98). This indicates that no severe multicollinearity exists among the variables, validating the stability of the logistic regression model.

**Table 4:** Multivariate analysis of invasion and metastasis in primary liver cancer.

Index	*B*	SE	Wald	*p*-value	OR	95% CI
Capsule intact	1.017	0.340	8.947	0.005	2.765	1.420~5.382
Tumor ≥ 5 cm	0.827	0.226	13.390	< 0.001	2.286	1.468~3.561
AFP	0.796	0.249	10.219	< 0.001	2.217	1.361~3.611
CAR	0.981	0.195	23.309	< 0.001	2.667	1.820~3.908
CD147	1.133	0.205	30.546	< 0.001	3.105	2.077~4.641
IL-6	1.027	0.314	10.697	< 0.001	2.793	1.510~5.165

### 3.5 Construction of the nomogram prediction model

**Figure 1:**
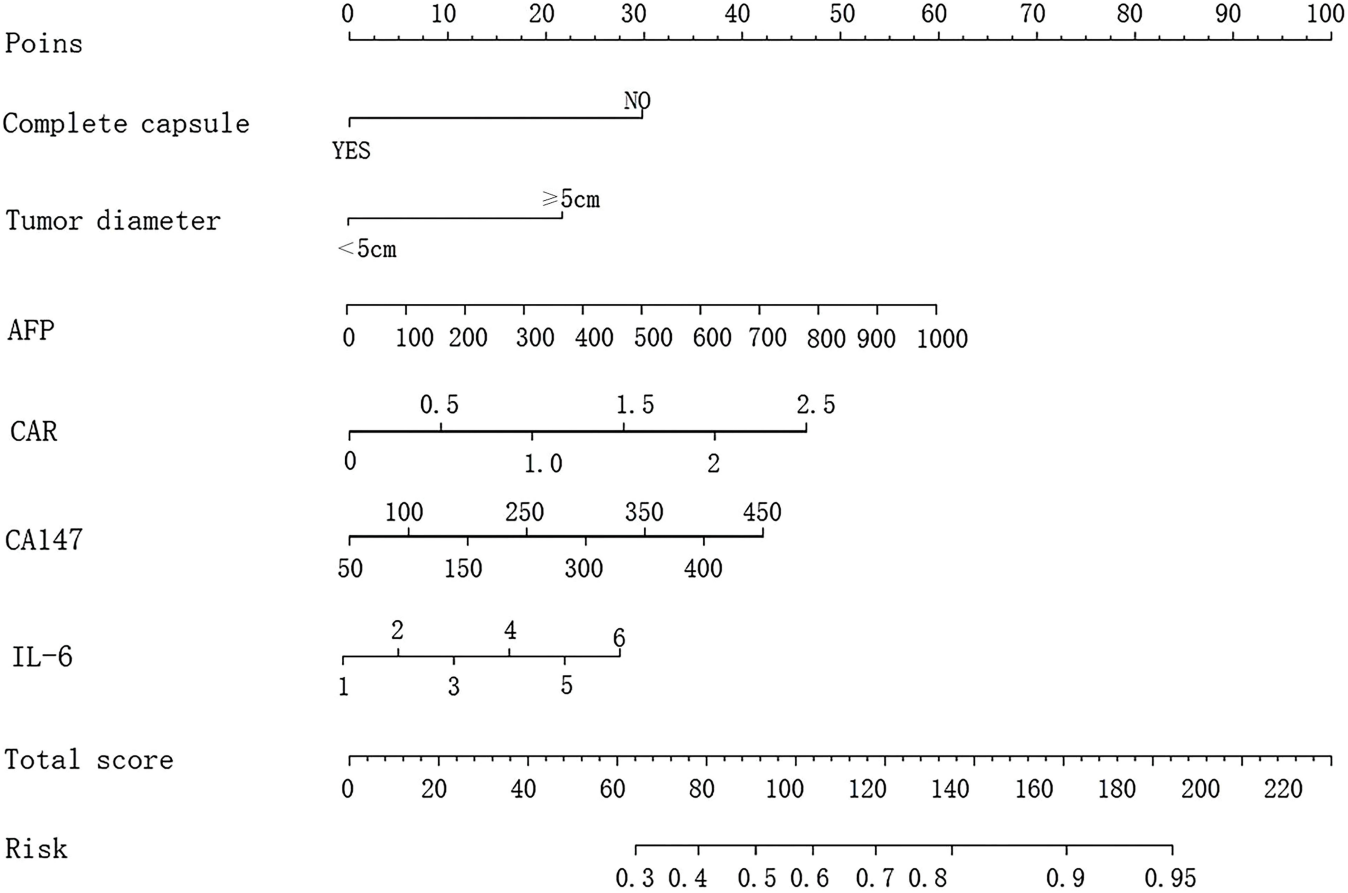
Nomogram model for predicting invasive metastasis of primary liver cancer based on six risk factors: complete capsule, tumor diameter, AFP, CAR, CD147, and IL-6.

The nomogram was constructed by converting the regression coefficients from the multivariate logistic regression model into scaled scores. Specifically, each independent variable’s regression coefficient was first standardized by dividing it by the maximum absolute coefficient to ensure that all variables contributed comparably to the total score. The scaled coefficients were then multiplied by a factor of 100 to convert them into integer scores (ranging from 0 to 100) for visual clarity on the nomogram. For example, the coefficient for CD147 (1.133) was scaled to 45 points, while IL-6 (1.027) was scaled to 41 points, based on their relative impact. The total score for each patient was the sum of all individual variable scores. This total score was then mapped to a probability scale using the logistic regression equation: Probability = 1/1 + *e* − (Intercept + ∑[Coefficient × Variable value]). This allowed visualization of the individual probability of invasion and metastasis based on combined risk factors (**Figure 1**).

### 3.6 Validation of the nomogram prediction model

**Figure 2:**
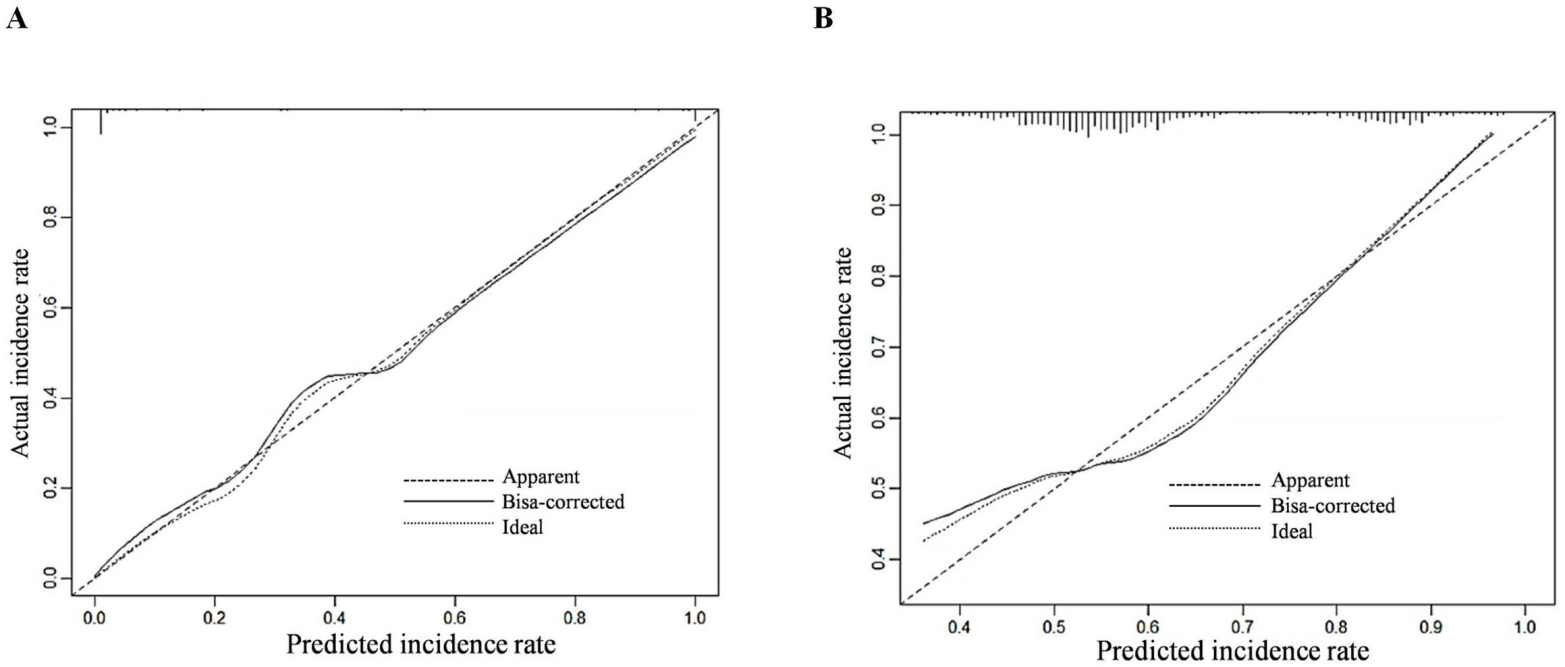
Calibration curves for predicting invasive metastasis of primary liver cancer using the nomogram model in the training **(A)** and validation **(B)** sets.

**Figure 3:**
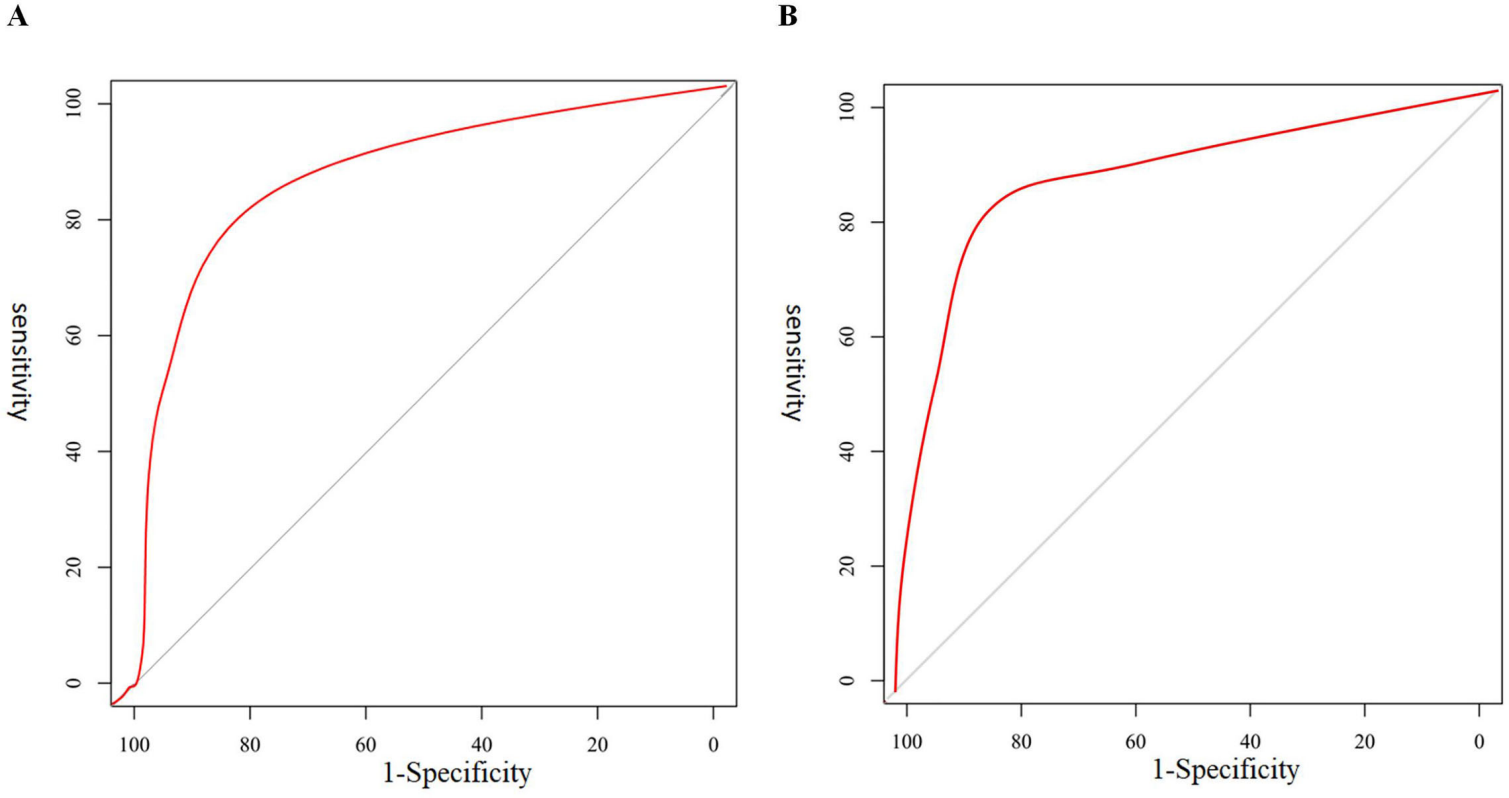
ROC curves for predicting invasive metastasis using the six-factor nomogram (complete capsule, tumor diameter, AFP, CAR, CD147, and IL-6) in the training **(A)** and validation **(B)** sets.

The C-index of the prediction model was calculated, with values of 0.884 (95% confidence interval [CI]: 0.738–0.932) for the training group and 0.841 (95% CI: 0.741–0.939) for the validation group. The calibration curves demonstrated good agreement between the predicted probability and the actual probability in both groups, without significant deviation, suggesting that the prediction model has good calibration (**Figure 2**). The AUC of the ROC analysis was 0.852 (95% CI: 0.824–0.979) for the training group and 0.839 (95% CI: 0.791–0.912) for the validation group, suggesting that the prediction model has good discrimination (**Figure 3**).

### 3.7 Decision curve analysis of the nomogram prediction model

**Figure 4:**
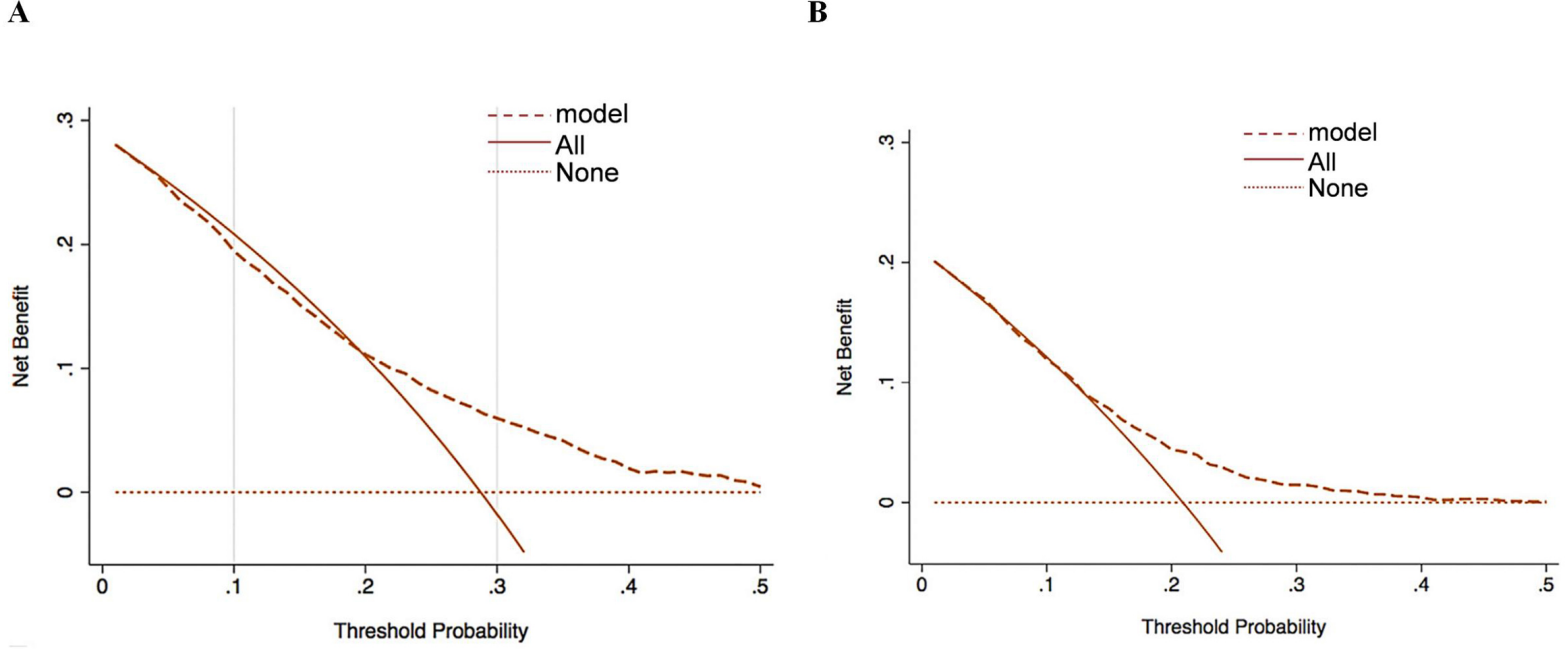
Decision curve analysis for clinical intervention based on the six-factor nomogram-predicted metastasis risk in the training **(A)** and validation **(B)** sets.

The applicability of the clinical prediction model was further assessed by DCA, with the horizontal axis indicating the threshold probability and the vertical axis indicating the net benefit. The results showed that using the constructed prediction model to predict invasive metastasis of primary cancers yielded high clinical benefit and strong applicability (**Figure 4**).

## 4 Discussion

Primary liver cancer is one of the most common malignant tumors in clinical practice. Hepatic resection, liver transplantation, and radiofrequency ablation are important methods for treating early-stage disease, but most patients are in the middle to late stage, or even the terminal stage, at the time of diagnosis, which may be related to the invasive metastasis of malignant tumors (12, 13). The etiology of primary hepatocellular carcinoma is complex and closely related to viral hepatitis, long-term alcohol abuse, fatty liver, and other factors. Currently, surgical resection, local ablation, and interventional therapy are the main therapeutic means, but the prognosis of patients is still poor, and invasive metastasis is the main cause of treatment failure and patient death. Therefore, it is necessary to establish a simple and highly accurate prediction model to assess the risk of invasive metastasis in patients with primary liver cancer at an early stage, to facilitate timely intervention and treatment.

The process of invasion and metastasis of liver cancer cells involves the regulation of multiple genes and signaling pathways, including those related to cell proliferation, adhesion, migration, and invasion. An in-depth study of the molecular mechanisms underlying the invasion and metastasis of liver cancer will help reveal the key factors involved in its occurrence and development, and provide new targets for clinical diagnosis and treatment. The results of this study showed that intact capsule, tumor > 5 cm, AFP, CAR, CD147, and IL-6 were all independent factors affecting the invasion and metastasis of primary liver cancer. Capsule formation is considered to be the result of chronic expansile growth and a barrier for tumor cell metastasis. Studies have found that the absence of a tumor capsule is related to regional lymph node metastasis and may be a factor affecting the prognosis of patients with liver cancer (5, 6). It has been found in the research on colorectal cancer and lung cancer that larger tumor size may be related to poor prognosis of patients, which may be due to a higher risk of microvascular invasion, making tumor patients more vulnerable to invasion and metastasis (14, 15). In a study of colorectal cancer, it was found that a higher AFP level had higher specificity (78.60%), sensitivity (88.00%), and accuracy (83.30%) in the diagnosis of liver metastasis in patients (16). It has been found that the serum CD147 level of ovarian cancer patients is significantly higher than that of patients with benign ovarian diseases and is associated with the prognosis of patients’ recurrence, which may be due to the fact that CD147 can activate matrix metalloproteinases (MMPs) and degrade the extracellular matrix of tumor cells, thus inducing invasion and metastasis of tumor cells and then promoting the recurrence of tumors (17). In one study, by comparing the level of CAR in cancer patients with different prognostic status, it was found that the level of CAR in patients with a poor prognosis was significantly elevated, and its level was negatively correlated with the patients’ survival time (18). It has been found that serum IL-6 shows an elevated level in a variety of malignant tumors, which may play a role in the development of tumors by affecting the survival, proliferation, and angiogenesis of tumor cells (19). IL-6 can promote angiogenesis in the tumor microenvironment and provide more nutrition and oxygen support for liver cancer cells, which is conducive to their growth and metastasis. IL-6 can also inhibit the activity of immune cells, making it easier for liver cancer cells to escape from the immune monitoring and removal of the body, thereby accelerating the process of invasion and metastasis (20). Current multibiomarker algorithms (e.g., GALAD) significantly improve early HCC diagnosis but focus primarily on tumor detection rather than metastasis prediction. Our model addresses a critical unmet need by specifically targeting invasion/metastasis risk using CD147 and IL-6 biomarkers, mechanistically linked to extracellular matrix remodeling (CD147) and prometastatic inflammation (IL-6). Integrating these novel markers with established factors (AFP, CAR) achieved an AUC of 0.852, demonstrating potential synergy with existing frameworks. For instance, CD147 may complement DCP (a GALAD component) in vascular invasion prediction, while IL-6 could refine risk stratification in AFP-negative subgroups. Future studies should explore hybrid models that dynamically incorporate imaging, traditional biomarkers (AFP/DCP), and emerging markers (CD147/IL-6) to optimize personalized surveillance and intervention (21). In addition, autophagy is an intracellular lysosome-dependent degradation system that is closely related to the occurrence and development of tumors. IL-6 may promote the chemotherapy resistance and malignant behavior of liver cancer cells by activating the autophagy pathway (22). According to the above results, it is speculated that the observation of various laboratory indicators in patients with primary liver cancer may play an important role in the early diagnosis of invasion and metastasis of these patients.

Since there is no obvious special reaction in the early stage of cancer, the diagnostic accuracy of relying solely on a single method is low; therefore, the combination of multi-indicator tests may be beneficial for the diagnosis of invasive metastasis of primary liver cancer. In this study, a column-line diagram prediction model was established based on the relevant influencing factors, and it was found that the consistency index of the column-line diagram risk prediction model was 0.884. It was validated by the ROC curve, which revealed that the column-line diagram model had a high diagnostic efficacy for invasive metastasis of primary hepatocellular carcinoma. In patients with primary liver cancer, targeted therapeutic measures can be formulated based on relevant risk factors to adjust the treatment program, which is of great significance in improving the safety of treatment, reducing the risk of invasive metastasis, and improving the prognosis of patients. Although serum biomarkers like AFP, CD147, and IL-6 may be biologically correlated (e.g., inflammation and tumor progression pathways), the VIF test confirmed no severe multicollinearity (all VIF < 10), suggesting their independent contributions to the nomogram are statistically valid. This supports the reliability of the model despite potential biological interconnections.

The developed nomogram offers significant clinical utility by providing a quantitative, individualized tool to predict the risk of invasive metastasis in liver cancer patients. In clinical practice, this model could be integrated into treatment decision-making at multiple levels: (1) For high-risk patients, clinicians may consider more aggressive surveillance (e.g., shortened imaging intervals to 3 months) or adjuvant therapies (e.g., postoperative TACE or targeted therapy), even after curative resection; (2) For intermediate-risk patients, the model could help stratify eligibility for clinical trials evaluating novel adjuvant approaches; (3) For low-risk patients, it may support decisions to reduce overtreatment and minimize unnecessary interventions.

However, this study also has certain limitations. First, the retrospective design may introduce selection bias and unmeasured confounding factors, despite our rigorous statistical adjustments. Second, although internal validation demonstrated robust performance, external validation in multicenter cohorts with diverse ethnic populations and practice patterns is needed to confirm generalizability. Fourth, the dynamic nature of liver cancer management (e.g., evolving immunotherapy protocols) suggests our model may require periodic updates to maintain clinical relevance. Finally, while the nomogram incorporates key prognostic variables, additional biomarkers (e.g., ctDNA, inflammatory indices) could further enhance predictive accuracy in future iterations.

## 5 Conclusion

In summary, the nomogram prediction model based on serum CD147, IL-6, and other risk factors has high diagnostic value for the invasion and metastasis of primary liver cancer. However, this study has limitations, such as a small sample size, a single-center retrospective design, and the lack of external validation. Future studies should increase the sample size, conduct prospective research, and perform external validation to improve the accuracy of the model. Future research should incorporate additional clinicopathological factors to enhance the nomogram’s generalizability, such as Child–Pugh liver function grade (a critical indicator of hepatic reserve), vascular invasion (a key predictor of metastasis), and tumor differentiation grade (reflecting tumor malignancy). These factors, widely recognized in clinical decision-making, may further refine the model’s predictive accuracy across diverse patient populations.

## Data Availability

The raw data supporting the conclusions of this article will be made available by the authors, without undue reservation.
